# EMMNet: Sensor Networking for Electricity Meter Monitoring

**DOI:** 10.3390/s100706307

**Published:** 2010-06-24

**Authors:** Zhi-Ting Lin, Jie Zheng, Yu-Sheng Ji, Bao-Hua Zhao, Yu-Gui Qu, Xu-Dong Huang, Xiu-Fang Jiang

**Affiliations:** 1 School of Computer Science and Technology, University of Science and Technology of China, Hefei, Anhui 230027, China; E-Mails: zhengms@mail.ustc.edu.cn (J.Z.); bhzhao@ustc.edu.cn (B.-H.Z.); ygqu@ustc.edu.cn (Y.-G.Q.); suphone@mail.ustc.edu.cn (X.-F.J.); 2 The State Key Laboratory of Networking and Switching Technology, Beijing, 100876, China; 3 National Institute of Informatics, Tokyo, 101-8430, Japan; E-Mail: kei@nii.ac.jp (Y.-S.J.); 4 Hangao Electronics Co., Ltd. Anhui, China; E-Mail: huangxudong1227@163.com (X.-D.H.)

**Keywords:** smart sensor, electricity meter monitoring, dynamic tree protocol, application

## Abstract

Smart sensors are emerging as a promising technology for a large number of application domains. This paper presents a collection of requirements and guidelines that serve as a basis for a general smart sensor architecture to monitor electricity meters. It also presents an electricity meter monitoring network, named EMMNet, comprised of data collectors, data concentrators, hand-held devices, a centralized server, and clients. EMMNet provides long-distance communication capabilities, which make it suitable suitable for complex urban environments. In addition, the operational cost of EMMNet is low, compared with other existing remote meter monitoring systems based on GPRS. A new dynamic tree protocol based on the application requirements which can significantly improve the reliability of the network is also proposed. We are currently conducting tests on five networks and investigating network problems for further improvements. Evaluation results indicate that EMMNet enhances the efficiency and accuracy in the reading, recording, and calibration of electricity meters.

## 1. Introduction

Power line networking devices were developed decades ago for automatic electricity-meter monitoring. Any electronic device connected to an electrical power line can use this technology for data communications without additional wiring. However, this use remains on the sidelines due to problems with quality of service, low data rates, range limitations, interoperability, and high cost. Recently, industries have provided remote reading for electricity meters via wireless connectivity, such as CDMA, GPRS, and PHS, using various radio spectra. These solutions are generally limited to peer-to-peer and one-level communication architectures [1]. When subjected to different user environments, they face various challenges. Therefore, we have developed an electricity meter monitoring network (EMMNet) based on heterogeneous smart sensor networks.

According to the classification given by Rivera *et al*. a smart sensor includes certain functionalities such as processing, communication and integration [2]. The smart sensor is emerging as a promising technology in a large number of application domains. For instance, Granados *et al*. [3] developed a smart sensor for real-time high-resolution frequency estimation in power systems. Their proposed smart sensor uses the chirp z-transform to compute the power spectrum and utilizes a commercially available current clamp, a Hall-effect sensor or a resistor as the primary sensor. Rodriguez-Donate *et al.* [4] presented a novel smart sensor to estimate motion dynamics, inclination, and vibration parameters on industrial manipulator robot links based on two primary sensors: an encoder and a triaxial accelerometer. Trejo-Hernandez *et al.* [5] developed a fused smart-sensor in order to improve the online quantitative estimation of flank-wear area in CNC machine inserts, from the information provided by primary sensors such as the monitoring current output of a servoamplifier and an accelerometer. Son *et al.* [6] developed a smart sensor system to acquire three types of signals involving vibration, current, and flux from induction motors. This system consisted of four modules: sensor, interface, server, and fault diagnosis module. The authors claimed that the smart sensor system can replace expensive traditional sensors for fault testing of induction motors.

In most applications, a smart sensor node is expected to acquire some physical measurements, perform local processing and storage, and communicate within a short distance [7–9]. The ability to communicate not only allows information and control to be communicated across the network, but also enables nodes to cooperate in performing more complex tasks, such as statistical sampling, data aggregation, and system status monitoring [10].

Although sensor network platforms already exist, for electricity meter monitoring we have to design an entirely new system because a meter-monitoring scenario has specific requirements. First, wireless access is very challenging in urban environments, where electricity meters in tall buildings are often installed in a metal chest and separated by concrete walls. Most of the existing platforms can only provide very short-distance communication in this environment. We find that they cannot satisfy our requirements, through field measurement. Second, none of the existing platforms conform to the China National Standard for automatic electricity-meter monitoring. The radio frequency band is specifically designated to 470–510 MHz [11]. Finally, the nodes, which are called data collectors in the automatic electricity meter-reading field, need to be connected to electricity meters. Thus, we should consider safety factors in the node layout. In light of such needs, we present EMMNet, an integrated sensor environment for remote electricity meter monitoring. EMMNet is composed of data collectors, data concentrators, hand-held devices, a centralized server, and clients. The novelty of this work is the development of a smart sensor for real-time electricity meter monitoring. In addition, our EMMNet has following features compared with other existing remote meter monitoring systems:

- Dedicated design of radio-frequency (RF) circuit. Application of automatic electricity meter monitoring faces various challenges, such as tall buildings in urban areas, long distances in suburban regions, and signal interference. However, the indoor communication range of the most typical sensor network platforms is about 25 m and this distance cannot meet the requirements of electricity meter monitoring. Therefore, we added broadband, fixed-gain, linear amplifiers, and low noise amplifiers to the data collectors and the data concentrators. A data concentrator is equivalent to a base station or gateway in sensor networks.
- High reliability and performance. Some existing automatic meter monitoring systems utilize ZigBee [12], which is a specification for a suite of high level communication protocols for wireless personal area networks. But ZigBee can only operate in three radio bands, which limits its applications. A high-performance dynamic tree (DT) protocol is proposed in this paper. It is based on CSMA/CA and has no frequency limit. Even if some nodes (data collectors) lose their connection frequently because of environmental changes, other nodes can quickly repair the routes.
- Low operational cost. Compared with other existing remote meter monitoring systems based on GPRS [13], the operational cost of EMMNet is relatively low because only the data concentrators incur in costs for using GSM/GPRS network.
- Changeability. China Power System is currently being reconstructed, and the requirements of the automatic electricity-meter-reading system change from time to time. Data collectors and data concentrators in EMMNet support wireless reprogramming, which can satisfy customer requirements in a timely manner.

## 2. Existing Sensor Network Platforms

Recently, a number of sensor network platforms have been proposed. Table 1 compares the prices and features of our EMMNet and some typical systems. The MICA2 mote is a third-generation mote module used for enabling low-power wireless sensor networks. It is supported by the MoteWorks wireless sensor network platform for reliable *ad hoc* mesh networking [14]. The TelosB mote is an open source platform designed to enable cutting-edge experimentation for the research community. TelosB bundles all the essentials for lab studies into a single platform, including USB programming capability, an IEEE 802.15.4 radio with integrated antenna, a low-power microprogrammed control unit (MCU) with extended memory, and an optional sensor suite (TPR2420) [15]. The MICAZ is also a 2.4-GHz mote module based on the Atmel ATmega128L [16]. Unlike most existing platforms, the Imote2 contains the Intel PXA271 CPU, which can operate at 13–416 MHz with dynamic voltage scaling [17]. Building on the highly successful Tmote Sky, Moteiv introduced Tmote Mini, the latest generation of wireless sensor network hardware. Moteiv’s Tmote Sky and Tmote Mini platforms both combine Texas Instruments MSP430 microcontrollers with TI/Chipcon low-power radios [18,19]. In addition, BTnode is a versatile, autonomous wireless communication and computing platform based on a Bluetooth radio, low-power radio, and microcontroller. The low-power radio is the same as that used on the Berkeley MICA2 mote, making BTnode a twin of MICA2 [20].

As shown in Table 1, among all these platforms the smart sensor in EMMNet provides the longest communication distance. It is thus suitable for applications in complex urban environments, where electricity meters in tall buildings are often installed inside metal chests and separated by concrete walls. The external memory space of the data collector in EMMNet is 4 MB and that of data concentrators is 8 MB. The external memory space of EMMNet is large, because collectors and concentrators need to record the data and parameters from hundreds of electricity meters. The cost of EMMNet is also lower than that of any other platform listed in Table 1. A dynamic power-management technique is an additonal feature of our system. This technique dynamically reconfigures the system to provide the requested services. The current draw of the processor is 8 mA in active mode, and it drops to about 30 uA while in sleep mode.

## 3. System Architecture

**Figure 1.**
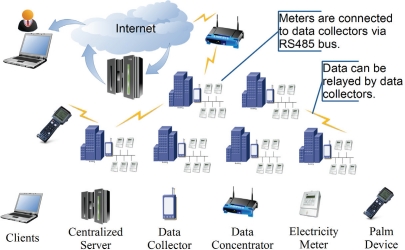
Architecture of EMMNet.

EMMNet is built on a heterogeneous networking infrastructure. In the automatic electricity meter-reading field, a sensor node is usually called a data collector, and a base station or gateway in the sensor network is called a data concentrator. EMMNet involves data collection, transmission, and access phases, as shown in Figure 1.

To obtain average real power information the electricity meter utilizes an ADE7755, which is a high accuracy electrical energy measurement sensor. The error of an ADE7755 is less than 0.1% over a dynamic range of 500 to 1. The only analog circuitry used in an ADE7755 is in the ADCs and reference circuit. All other signal processing (e.g., multiplication and filtering) is carried out in the digital domain. This approach provides superior stability and accuracy over extremes in environmental conditions and over time. We connect electricity meters to data collectors via an RS485 bus. Electricity meters are installed in a metal chest, and data collectors are usually beside them for safety purposes. The sensor data in our system flow from the data collectors to a data concentrator over the DT protocol. Data can also be relayed via data collectors if the electricity meters are far from the data concentrator. The data concentrators transmit electricity meter data to the remote centralized server that provides data logging. The data is transmitted using the TCP/IP suite of protocols and therefore can be carried over types of many networks, including LAN, CDMA, and GPRS. We also propose a back-end client/server architecture to provide a user interface to the system and support further centralized processing for higher-level applications. The GUI visualization component has a graphical display that enables an overall view of the network to be shown. The visualization component also has a relation layer to display relationships between nodes and a node layer to draw the nodes themselves. Customers can access their electricity bills whenever necessary, and an administrator can check the electricity meters’ status and modify the parameters of each electricity meter if needed. Hand-held devices are also included and used as “mobile data concentrators” in the EMMNet. If any fault occurs in the fixed data concentrators, the hand-held devices can still be used to collect data in a timely manner. Hand-held devices can communicate with other devices in four different ways: 470 MHz wireless channel, IrDA, RS232, and RS485. In addition, data collectors and data concentrators can be reprogrammed by hand-held devices if the requirements of the automatic electricity-meter-reading change.

## 4. Hardware Design

EMMNet is composed of data collectors, data concentrators, hand-held devices, a centralized server, and clients. The hardware design of smart sensors will be detailed in subsequent subsections.

### 4.1. Data Collector

**Figure 2.**
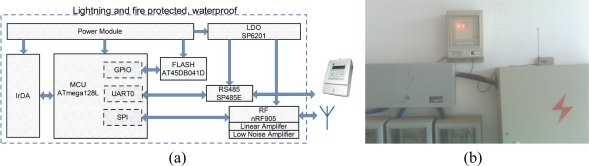
(a) Hardware architecture of a data collector. (b) Spot photo of data collector.

Each node, also called a data collector or collector, has a microprocessor, serial interface flash memory, RF transceiver, linear amplifier, low-noise amplifier, RS-485 transceiver, and IrDA module. The basic hardware structure schematic and a photo of the data collector are shown in Figure 2.

*Microprocessor*: Usually, one of the three microcontrollers, *i.e*., ATmega128L, MSP430, and Silicon Labs C8051, is used in nodes of the sensor network. After intensive investigations, we chose the ATmega128L microcontroller for our EMMNet platform, because it is a low-power microcontroller based on the AVR-enhanced Reduced Instruction-Set Computer (RISC) architecture. The ATmega128L provides the following features: 128 K bytes of in-system programmable flash with read-while-write capabilities, 4 K bytes Electrically Erasable Programmable Read-Only Memory (EEPROM), 4 K bytes static RAM (SRAM), and 53 general purpose IOs. Making rational use of the microprocessor resources, we leave ample room for expansion. At present, we have used 62.00% of the port, 34.87% of the code memory, 72.75% of the data memory and 88.23% of the EEPROM. Ports assignment is listed in Table 2.

*RF Transceiver*: We use the Nordic nRF905 transceiver because its carrier frequencies are suitable for automatic electricity meter monitoring and there is no associated proprietary protocol. Its current consumption is very low: in transmit mode, only 9 mA at an output power of −10 dBm, and in receive mode, 12.5 mA. However, EMMNet faces various challenges. Therefore, we add a broadband, fixed-gain, linear amplifier and a low-noise amplifier to the RF circuit.

*Memory*: A memory bank with relatively large capacity is believed to be beneficial for data storage and buffering in EMMNet since there can be up to 100 electricity meters connected to a single data collector. Therefore, we chose the AT45DB041D, a 4-Mbit serial-interface flash memory. In addition to the main memory, the AT45DB041D also contains two SRAM buffers of 264 bytes each. In our design, we take full advantage of this external SRAM, greatly easing the problem of small SRAM in the ATmega128L.

### 4.2. Data Concentrator

**Figure 3.**
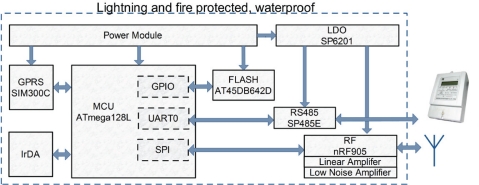
Hardware architecture of a data concentrator.

The key components of the data concentrator are similar to those of the data collector. These devices can share most of the drivers and application codes, which greatly reduces the difficulty of our design and of debugging. However, the data concentrator requires more external memory because it is responsible for transmitting electricity meter data from hundreds of data collectors to the remote centralized server. A GPRS module is also added to the design of the data concentrator. Modularity is a key tenet of our design, reflected in the separation of the GPRS module and the 470 MHz wireless module on the data concentrator. The basic hardware structure is illustrated in Figure 3.

*Memory*: We use the AT45DB642D instead of the AT45DB041D. Its 69,206,016 bits of memory are organized as 8,192 pages. In addition to the main memory, the AT45DB642D also contains two SRAM buffers of 1,056 bytes each.

*GPRS module*: We chose the SIM300C for the data concentrator. The SIM300C is a tri-band GSM/GPRS engine that works in the EGSM 900 MHz, DCS 1,800 MHz, and PCS 1,900 MHz frequencies. With a tiny configuration of 50 × 33 × 6.2 mm, SIM300C meets the space requirements in our industrial application. The SIM300C is designed to have power-saving features: the current consumption is as low as 2.5 mA in sleep mode.

### 4.3. Memory Arrangement

The ATmega128L contains 128 K bytes of in-system programmable flash with read-while-write capabilities, 4 K bytes EEPROM, and 4 K bytes SRAM. The in-system programmable flash, which is used for storing program code, does not cause a bottleneck in our design. The problem is that the SRAM is relatively insufficient. It is used for temporary storage of data, produced as a result of processing, until instructions call for the data to be used again in subsequent processing. In addition, during interrupts and subroutine calls, the return address is stored on the stack. The stack is effectively allocated in the general data SRAM, and consequently the stack size is only limited by the total SRAM size and the usage of the SRAM. Therefore, we have to reserve enough SRAM space. As mentioned before, the AT45DB041D contains two SRAM buffers of 264 bytes each, and the AT45DB642D also contains two SRAM buffers of 1,056 bytes each. Therefore, we can take full advantage of these parts of the space and treat them as external SRAM buffers. Thus, the available SRAM of the collector is increased by 12.89%, and that of the concentrator is increased by 50.10%. EEPROM in the ATmega128L is used for storing the device parameters and the routing table. Since each entry of the routing table occupies 5 bytes, the EEPROM can record about 800 entries, which is sufficient for this type of application. The external flash is a type of non-volatile storage that can be electrically erased and reprogrammed. No power is needed to maintain the information stored in the chip. Therefore, all the data obtained from the electricity meters are kept in external flash. The data concentrator is required to record hourly electricity meter data in last fifteen days and daily electricity meter data in last sixty days. And the flash memory size used in a concentrator is 69,206,016 bits, so a single data concentrator can monitor at most 2,300 electricity meters, which is sufficient for the application of the automatic electricity meter monitoring.

### 4.4. Energy Consumption

Minimizing the energy consumption of our platform is essential, so a dynamic power-management technique is a feature of our system. This technique dynamically reconfigures the system to provide the requested services and performance levels with a minimum number of active components or a minimum activity level on such components. We chose the SP6201 to dynamically control the RF module. The SP6201 is CMOS low dropout regulator (LDO), which offers an extremely low quiescent current. The current of the nRF905 and the SP485E is zero in the sleep mode using the SP6201. Table 3 shows the average current of a data collector under different modes.

### 4.5. Protection Measure

The design of an automatic electricity monitoring system needs to consider lightning and fire protection as well as waterproofness. Therefore, transient voltage suppressors are added to protect devices from damage due to lightning strikes by intercepting such strikes and safely passing their extremely high voltage. Thermistors are also included in our design. A thermistor is a type of resistor whose resistance varies with temperature. Thermistors are widely used as inrush current limiters, temperature sensors, overcurrent protectors, and self-regulating heating elements. In addition, waterproof sealing is also needed for collectors and concentrators.

## 5. Dynamic Tree (DT) Protocol

Many intricate protocols of sensor networks have been proposed based on laboratory settings in the past few years. However, a significant gap remains between the laboratory settings and the application environments. In this section, we present a dynamic tree (DT) protocol that is designed for smart sensors in harsh environments. We also discuss problems that are hard to be modeled and simulated in laboratory settings and how to improve the reliability of the network in a real scenario.

### 5.1. Network Initiation

Initially, the *DtA* value of each data collector equals *DtA_Max* + 1, where *DtA* indicates the hop count to the data concentrator*,* and *DtA_Max* is the maximum depth of the EMMNet. The *DtA* value of the data concentrator is set to 0. When the collector is opened, it will regularly send a request message, RM. This request message includes two fields: *packet_sender_ID* and *packet_DtA*. Here, *packet_sender_ID* indicates who sends the message, and *packet_DtA* describes the hop count between the sender and the concentrator. When any collector receives an RM packet, it can reply if its *DtA* is smaller than the *packet_DtA* in RM and does not equals *DtA_Max*. Therefore, only the data concentrator can reply to collector requests at the beginning. The reply to the request message, RRM, has the following fields: *packet_source_ID*, *packet_DtA*, and *packet_sender_ID*. Here, *packet_source_ID* denotes the ID of the concentrator.

If any collector receives an RRM and its *DtA* equals *DtA_Max* + 1, its *DtA* is set to *packet_DtA* + 1 no matter what the *packet_DtA* value is. Meanwhile, its *father_ID* list and *root_ID* are updated according to *packet_sender_ID* and *packet_source_ID*, where *father_ID* list and *root_ID* are used for recording the potential relay nodes and the concentrator ID.

If a collector receives another RRM and its *DtA* is smaller than *DtA_Max* + 1, it should make judgments based on the value of *packet_DtA* and its own *DtA*. If *packet_DtA* + 1<*DtA*, the value of *DtA* and *root _ID* should be updated. Meanwhile, it should clear the *father_ID* list before inserting *packet_sender_ID* as a new relay node. If (1) *packet_DtA* + 1=*DtA*, (2) *packet_source_ID* equals *root _ID*, and (3) *packet_sender_ID* is not in the *father_ID* list, *packet_sender_ID* is added to the end of the *father_ID* list.

In the electricity meter monitoring application, we not only collect the quantity of electric, but also control the parameters of the electricity meters. Transmission is divided into uplink and downlink channels. The uplink channel is used for transmission of information from the collectors to the concentrator. The downlink channel is used for transmission of information from the concentrator to the collectors, including resetting the sample ratio, altering the collection parameter, and sending query commands. Therefore, we should establish the downlink channel. Each collector needs to transmit a routing message to the concentrator when its *DtA* is smaller than *DtA_Max* + 1. If a collector receives routing messages from its child collectors, the routing information is recorded in the routing table and the messages are relayed to its parent collectors.

**Figure 4.**
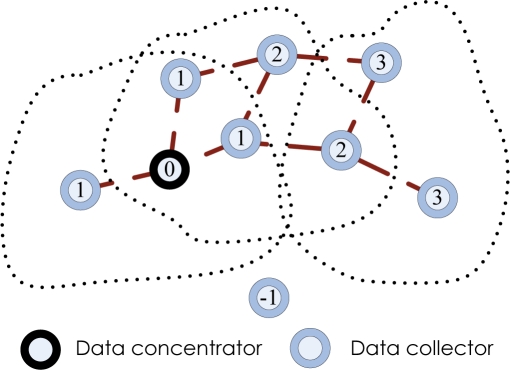
Structure of EMMNet with eight collectors.

The structure of the EMMNet after network initiation is shown in Figure 4. Each device has a communication range, which is illustrated by the dotted line. The communication range is not a perfect circle due to the complicated communication environment. The dashed lines indicate relationships among the collectors and the concentrator. The numbers in the circles indicate the value of *DtA*. The node whose *DtA* value equals zero denotes the data concentrator. A collector whose *DtA* value is larger than zero at least has a parent collector, and the *DtA* value of this collector is larger than that of its parent collector by one.

### 5.2. Data Transmission

Every fifteen minutes, collectors report electricity meter data to the concentrator. However, most of the time collectors are in sleep mode. The concentrator transmits the control message to collectors according to the routing table, while collectors report the data based on the *father_ID* list. If a child collector detects that one of its parent collectors does not respond, it can choose another spare parent collector to relay the message. If all of its parent collectors fail to respond—the situation may be caused by network structure change—it should rejoin the network. In other words, this collector needs to reset *DtA* to *DtA_Max* + 1 and send request messages, RM. If a collector receives an RM from its parent collector and its *packet_DtA* does not equal *DtA*-1, it needs to remove the corresponding item from the *father_ID*. A special case is when the *father_ID* list contains only one single item: the collector should also rejoin the network. Making use of this strategy, EMMNet can maintain connectivity without frequent network reconstruction, even if some collectors die because of non-recoverable errors or malfunction.

### 5.3. Analysis of DT Protocol

We analyze the DT protocol under the ideal case and discuss the problems occurring in practical applications in this section.

**Figure 5.**
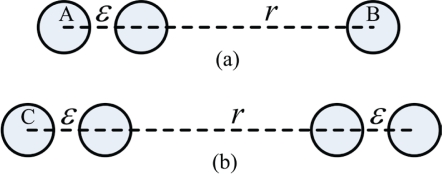
(a) Two hops require at least an interval of *r* + *ε*. (b) Three hops require at least an interval of *r* + *2ε*.

First, we make several assumptions to simplify the analysis:

1. The collectors in the EMMNet are distributed as a homogeneous spatial Poisson process□ in two-dimensional space.
2. All collectors and concentrators transmit at the same power level and have the same radio range *r*.
3. A distance of *d* between any collector and its concentrator is equivalent to [d/(0.75 r)] hops. This can be explained as follows: as shown in Figure 5, two hops require at least an interval of *r + ε*, and three hops require at least an interval of *r + 2ε*, where ε is an arbitrary small value.

This can then be extended to the situation of 2*n* hops by concatenating *n* 2-hop topological graphs. In the same way, 2*n* + 1 hops can be extended by concatenating *n* − 1 2-hop topological graphs and one 3-hop topological graph. We can conclude that 2n-hop or 2*n* + 1-hop requires at least an interval of *nr*. Thus, with the fact that 2 *n* hops require at most an interval of 2*nr*, it could be deduced that on average there are [d/(0.75 r)] hops between any collector and its concentrator.

(4) Taking into account the short distance in one hop, we use the free space channel model for estimating the radio hardware energy dissipation:
  (1) $$E_{TX}=E_{\mathrm{elec}}k+e_{\mathrm{fs}}d^{2}k$$

To receive this message, the radio expends:

(2) $$E_{\mathrm{RX}}=E_{\mathrm{elec}}k$$

The electronics energy (*E_elec_*) depends on such factors as digital coding, modulation, and filtering. The variable *d* indicates the distance to the receiver. The amplifier energy, e_fs_, depends on the distance *d* and the acceptable bit-error rate [21]. *k* is the length of the message.

According to the results in Reference [22], the expectation value of the total length of all segments connecting the collectors to the concentrator, L_v_, is given by:

(3) $$E\left[L_{v}\right]\approx \frac{1}{2n^{3/2}}$$

where *n* is the number of collectors. With this formula and the third assumption mentioned above, we can calculate the number of hops, *J*, in an EMMNet as:

(4) $$J=\frac{E\left[L_{v}\right]}{r\times 3/4}=\frac{2}{3n^{3/2}r}$$

The energy consumption of the communication between the collectors and the concentrator, *C*, can be evaluated as:

(5) $$E\left[C\right]=J\left[E_{\mathrm{TX}}+E_{\mathrm{RX}}\right]=\mathrm{Jk}\left[\left(E_{\mathrm{elec}}+e_{\mathrm{fs}}r^{2}\right)+E_{\mathrm{elec}}\right]$$

*E*[*C*] is minimized by the value of *r* that is a solution of:

(6) $$-\frac{2E_{\mathrm{elec}}}{r^{2}}+e_{\mathrm{fs}}=0$$

This equation shows how to maximize the performance of the sensor network. However, a significant gap remains between the assumptions and the application environment. Certain problems may arise in a practical application:

1. In the conventional literature on sensor networks, protocols are designed on the assumption of reciprocity between uplink and downlink channels. However, affected by the imbalance of the RF circuitries in practice, the reciprocity between the uplink and downlink channels cannot be maintained, which will cause a performance decrease.
2. It used to be widely believed that sensor networks with smaller average hop counts perform better than those with larger average hop counts. Nevertheless, we find out that when a collector chooses a parent collector with a much smaller *DtA* value, it may actually reduce the transmission success rate. That is because the child and parent nodes are too far from each other and the bit error rate is relatively high.
3. In the theoretical analysis, we ignore the fact that packet length affects the transmission success rate. Therefore, even if nodes can communicate with others without any trouble in the process of constructing the network, they may need to retransmit many times before the concentrator successfully receives the monitoring data. The reason is that the size of the monitoring data is usually much larger than that of RRM or RM.

We set the packet length of RRM and RM to equal that of the longest data packet to solve the above problems. The collector can only broadcast the RM a limited number of times, marked as *LT*, when the value of *DtA* is smaller than *DtA_Max* + 1. Evaluation results of the DT protocol from several applications show that our strategy greatly improves the performance of the EMMNets.

## 6. EMMNet Validation

### 6.1. Experiment Environment

The experiment environment for the proposed smart sensors is shown in this section. Collaborating with Hangao Electronics Co., Ltd., we deployed our systems in two cities and tested our systems over a three-month period. The data of 2024 electricity meters were +collected. All the devices of the test networks are listed in Table 4 and the elements used in EMMNet are listed in Table 5.

The interval between sending two RMs is 1 minute, the packet length of an RRM or RM equals 330 bytes, and the maximal depth of the EMMNet is 10 in these EMMNets.

**Figure 6.**
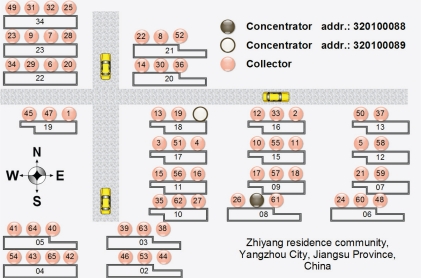
Two EMMNets deployed in Zhiyang residence community, Yangzhou City, Jiangsu Province, China.

Figure 6 shows the architectural plans of the Zhiyang residence community, Yangzhou City, Jiangsu Province, China. We deployed two of our systems in this community. The numbers in circles indicate the last two digits of the collector addresses.

### 6.2. Results

In our original design, there was no limit to the number of times a RM could be sent, even when the collectors had joined the EMMNets. A collector had a great chance of selecting a relay node with a smaller *DtA* value, when it constantly sent RMs. However, the performance of such relay nodes might be poor. We found that the transmission success rate decreased if a collector chose a parent collector with a very small *DtA* value. Therefore, we limited the number of broadcast packets when the value of *DtA* is smaller than *DtA_Max* + 1.

**Figure 7.**
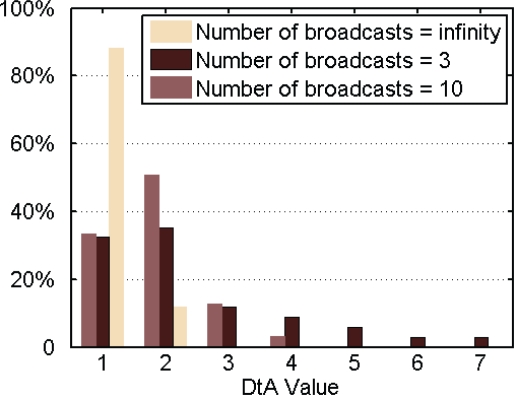
Distribution of *DtA* value in three different cases.

**Figure 8.**
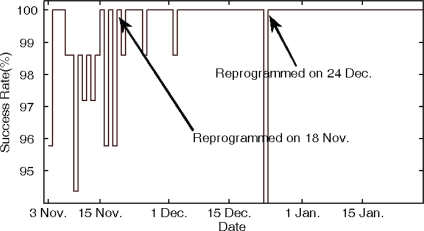
Average transmission success rate. System tested for three months; collectors reprogrammed twice during this period.

**Figure 9.**
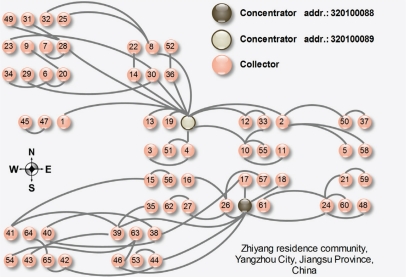
Network structures of EMMNets deployed in Zhiyang residence community.

The distribution of the *DtA* value is shown in Figure 7, and the transmission success rate is illustrated in Figure 8. From November 3–18, 2009, collectors could broadcast RMs without any limit. As shown in Figure 7, the network structure does not seem very good. All the *DtA* values of collectors are less than 3, and the average transmission success rate is relatively low. That is because child and parent nodes are too far from each other and the bit error rate is relatively high. Therefore, we reprogrammed the collectors with a hand-held device in the 470 MHz wireless channel. When the value of *DtA* was smaller than *DtA_Max* + 1, the collector could only broadcast the RM three times. On Deceber 24, 2009, we reprogrammed the devices and made the EMMNets dynamically choose the limited times that RM packets were broadcasted according to the transmission success rate. At first, the limited-times value *LT* was set to two; if the performance was poor, the *LT* was increased by two. Figure 8 shows that average transmission success rate is improved due to the advantage of the network topology. And Figure 9 plots the corresponding network structures of the EMMNets deployed in Zhiyang residence community. With the EMMNets, customers can obtain the energy flow amount at any time through internet and the maintainers can control the parameters of the electricity meters if necessary. The meter monitoring task can be done at the management department by using this system. What should be emphasized is that the operational cost of EMMNet is relatively low, compared with other existing remote meter monitoring systems based on GPRS. Meanwhile, EMMNets can realize automatic charge if they are connected to bank systems.

## 7. Conclusions

In this paper, we have presented a hierarchical smart sensor system for electricity meter monitoring. EMMNet is built on a heterogeneous networking infrastructure that includes data collectors, data concentrators, hand-held devices, a centralized server, and clients. The major challenge of adopting a sensor network to fulfill the requirements of electricity meter monitoring is the complicated environments, such as those with tall building structures, long distances, and signal interference. Our practical experience with sensor network deployment can provide guidance for the production of remote monitoring. We plan to extend the EMMNet to gas-meter and water-meter monitoring systems in the future. This work would be easily accomplished without changing the system a lot, because the main components of EMMNet are also suitable for these applications. And cameras may be added into data collectors in some applications. The major task of it is to compress images with low cost MCU.

## Figures and Tables

**Table 1.** Characteristic ubiquitous sensor network platforms.

**Processor**				**RF Transceiver**				
**Platforms**	**Program Space**	**External Space**	**Current Draw**	**Frequency**	**Current Draw**	**Indoor Range**	**Tx speed**	**Cost**
MICA2	128 kB	512 kB	8 mA	868/916 MHz	S: 27 mA at 5 dbm R: 10 mA	—	38.4 kbps	$125
TelosB	48 kB	1,024 kB	1.8 mA	2,400 MHz	S: — R: 23 mA	20–30m	250 kbps	$134
MICAZ	128 kB	512 kB	8 mA	2,400 MHz	S: 17.4 mA at 0 dbm R: 19.7 mA	20–30m	250 kbps	$99
Imote2	32 MB	32 MB	66 mA	2,400 MHz	—	<30 m	250 kbps	$299
Tmote Sky	48 kB	1,024 kB	2 mA	2,400 MHz	17 mA	—	250 kbps	$78
Tmote Mini	48 kB	1,024 kB	2 mA	2,400 MHz	17 mA	<30 m	250 kbps	—
Btnode	128 kB	128 kB	8 mA	433/868/916 MHz	S: 14.8 mA at 0 dbm R: 7.4 mA	20 m	76.8 kbps	$120
EMMNet	128 kB	4/8 MB	8 mA/30 uA	433/470/868/916 MHz	S: 20 mA at 6 dbm R:13 mA	180 m	50 kbps	$40

**Table 2.** Ports assignment of ATmega128L.

**Port**	**Function**
PB0-PB6	Connected to RF module
PD0-PD3	Connected to IrDA
PD4-PD7	LED
PE0-PE2	Connected to RS485 transceiver
PE3-PE7	Connected to RF module
PF0-PF3	Connected to external flash
PF4-PF7	JTAG test interface

**Table 3.** Average current of the data collector

**Chip**	**Tx current at 10 dbm**	**Rx current**	**Sleep**
ATmega128L	8 mA	8 mA	30 uA
nRF905	29 mA	13 mA	0
SP485E	1 mA	1 mA	0
AT45DB041D	25 uA	27 uA	25 uA

**Table 4.** Five EMMNets in two cities.

**Concentrator address**	**Num. of concentrator**	**Num. of hand-held devices**	**Num. of collectors**	**Num. of electricity meters**	**Location**
320100091	1	2	45	368	Huaian City, China
320100092	1	2	35	439	Huaian City, China
320100093	1	1	23	284	Huaian City, China
320100088	1	2	29	517	Yangzhou City, China
320100089	1	2	36	416	Yangzhou City, China

**Table 5.** Elements used in EMMNet.

**Data Collector**	**Data Concentrator**
ATMEGA128L	ATMEGA128L
ADL5320	ADL5320
nRF905	nRF905
sp6201	sp6201
PS2051	PS2051
--	SIM300C
AT45DB041D-SU	AT45DB642D
